# Socioeconomic Indicators That Matter for Population Health

**Published:** 2010-06-15

**Authors:** Paula M Lantz, Andrew Pritchard

**Affiliations:** Dept of Health Management and Policy, University of Michigan School of Public Health; University of Michigan School of Public Health, Ann Arbor, Michigan

## Abstract

Increasing research and policy attention is being given to how the socioeconomic environment influences health. This article discusses potential indicators or metrics regarding the socioeconomic environment that could play a role in an incentive-based system for population health. Given the state of the research regarding the influence of socioeconomic contextual variables on health outcomes, the state of data and metrics for these variables at the local level, and the potential for program and policy intervention, we recommend a set of metrics related to the socioeconomic composition of a community (including poverty, unemployment, and public assistance rates); educational attainment and achievement; racial segregation; and social-capital indicators such as density of voluntary organizations and voter turnout. These indicators reflect the evidence that population health gains depend on improvements in many of the fundamental social determinants of health, including meaningful employment, income security, educational opportunities, and engaged, active communities.

## Introduction

Increasing research and policy attention is being given to how the socioeconomic environment influences health (1,2). We define socioeconomic environment as a place with geographically defined boundaries that also has economic, educational, social, cultural, and political characteristics.

The socioeconomic environment shapes resources, opportunities, and exposures (positive and negative) (3). Theoretically, the neighborhood socioeconomic environment could influence health outcomes either directly or indirectly (1). Direct effects on health include injuries from crime or environmental hazards or illness from socially patterned toxic exposures. In addition, many aspects of the neighborhood socioeconomic environment — including poverty and discrimination — can be considered stressors. Chronic exposure to social stressors can elevate the body's stress response (via neural, euroendocrine, and immune systems) and produce "allostasis," a physiologic state that in the long run causes changes in the immune system and brain that can lead to disease through a variety of biological mechanisms (4,5). Other putative mechanisms linking socioeconomic environment and health are indirect, such as differential access to key resources like employment opportunities (which strongly influence income), food, housing, and health care services.

The degree to which these pathways play a role in producing contextual health outcomes is not well understood (6,7). Researchers encounter serious conceptual and methodological challenges to defining socioeconomic environments and in measuring contextual effects on health, especially over time (7-9). Nonetheless, research findings suggest that socioeconomic environment has a substantial effect on health risk behaviors (eg, tobacco use, poor diet, physical inactivity), health care use (eg, prenatal care, asthma care), and health outcomes (eg, functional health, cardiovascular disease, chronic disease mortality, and birth weight) (3,9-13).

Kindig has argued that financial incentives for the nonmedical determinants of health need to be developed (14), including the socioeconomic environment that shapes many aspects of our social, economic, and political lives. The purpose of this article is to identify a potential set of metrics regarding the socioeconomic environment that could play a key role in such a system. We used the following criteria to generate a set of metrics for this objective: 1) the indicator can be measured with reasonable validity and reliability across socioeconomic environments, 2) evidence is sufficient that the indicator is related to health outcomes and is amenable to program or policy intervention, and 3) measurement of the indicator could be used to create incentives for and measure progress toward population health goals.

## Indicators of the Socioeconomic Environment

Characteristics of a socioeconomic environment can be measured subjectively via individual self-reports, or objectively via direct observation or secondary data sources such as the census, administrative databases (eg, for crime, housing, education), or population-based surveys (2). Many of the indicators that researchers have considered in studies of socioeconomic environment and health have been included in individual community projects that attempt to define quality of life or community well-being in a particular area (2,15). In addition, many cities produce report cards or other documents that present metrics regarding the quality of life.

There is no consensus regarding which indicators of the socioeconomic environment are the most important determinants of population health. Nonetheless, there does appear to be a tacit acceptance that certain indicators have particular importance for mental and physical health. We focus on such indicators in 3 broad areas: community socioeconomic composition, social structure, and social cohesion/social capital.

### Community socioeconomic composition

The socioeconomic composition of a community is a crucial aspect of how context can shape individual health behaviors, exposures, and outcomes (1,16). Levels of education, employment, income, and income security in a community create and shape risks and benefits for health, many of which accumulate over the life course. Key indicators of the economic and educational composition of a community that can be considered individually and in combinations and that typically can be measured at multiple units of geography include 1) income, such as average household income and per capita income; 2) poverty rate, percentage of households receiving public assistance, and percentage of children receiving free or reduced lunch; 3) the unemployment rate and the percentage employed in professional or managerial occupations; 4) affordability of housing, homelessness rate, bankruptcy rate, foreclosure rate, and resident turnover rate; and 5) percentage of population aged 18 to 24 years with less than high school education, public high school dropout and graduation rates, percentage of third- and tenth-grade students at grade level in reading, and percentage of tenth-grade students at grade level in math.

The socioeconomic composition of a unit of geography (eg, census tract, zip code, county) could be measured using individual metrics or a set of metrics that together measure “community socioeconomic status.” Robert created a community socioeconomic disadvantage index at the census tract level by summing the following measures: percentage of households receiving public assistance, percentage of families earning less than $30,000 annually, and percentage of adult unemployment (16). Another approach is to conduct factor analysis or principal components analysis on a wide range of indicators to identify which ones combine to measure a latent concept that cannot be captured with a single indicator. For example, using data from their research on Chicago neighborhoods, Sampson and Morenoff created scales for 1) concentrated disadvantage (consisting of the percentage of families below the poverty line), percentage of families receiving public assistance, percentage of unemployed people in the labor force, and percentage of families headed by women; and 2) concentrated affluence (defined by the percentage of families with annual income higher than $75,000), percentage of adults with a college education, and percentage of adults employed in professional or managerial occupations (6,17). Another measure is the Index of Concentrations at the Extremes, which measures the proportional balance or imbalance of familial poverty and affluence in a neighborhood (18).

### Social structure

Several researchers have investigated the influence of social structure — the ways in which social institutions and embedded norms shape the behavior and experiences of social actors — on health outcomes (1). In particular, 3 aspects of the social structure have received substantial attention in health-related research: income inequality, racial segregation, and discrimination. The quantitative evidence for the effect of these social structural phenomena on health is mixed and faces serious methodologic challenges (1).

A growing body of research suggests that in both developing and developed countries the degree of inequality in the income distribution of a geographic area is associated with mortality (19,20). In addition, several studies have shown an association between the degree of racial segregation in a geographic area and mortality as well as other health outcomes (17,21,22). However, association is not causation; the mechanisms by which income inequality and segregation might lead to poor health outcomes are unclear. The role of relative versus absolute deprivation in producing health inequalities and whether any part of the association between income inequality and health outcomes is causal is debated.

Discrimination is difficult to observe or measure. It is typically measured as "perceived discrimination" via self-reported survey data. Self-reports of perceived discrimination or unfair treatment because of race or ethnicity have also been associated with some negative health outcomes in several studies (23,24). The proposed health mechanisms are both direct (denial of needed services/resources related to health) and indirect (increased psychosocial stress, increased health risk behavior as a coping mechanism).

### Social cohesion and social capital

Social integration, social networks, and social support — all of which have to do with the degree to which people are interconnected and embedded within social environments — are considered key to health (25). Many aspects of social relationships that combine and emerge at a collective level can also affect health. Social cohesion is the "extent of connectedness and solidarity among groups in society" (26) or the degree of trust, familiarity, values, and network ties shared among groups (including neighborhoods). Although debate continues, social capital generally refers to the social resources and benefits that emerge from strong social ties or social cohesion and facilitate collective action (26,27). Strong social ties and cohesion may create social capital or private and public resources that matter for health.

Several studies have linked measures of social cohesion and social capital to health-related behaviors or health status outcomes (25-29). Nonetheless, given that approaches to defining and measuring social cohesion and social capital vary greatly, comparisons across studies are hampered. In addition, the exact mechanisms by which social cohesion, social capital, or both may produce better health outcomes are unknown.

Social cohesion has been measured as the magnitude of social and economic divisions in a community in terms of the degree of racial segregation and income equality. Social cohesion has also been measured with survey items intended to measure social networks or to capture interpersonal trust (ie, the extent to which people in a neighborhood trust each other, get along, share values, and are willing to help each other). Social capital also has been measured as the level of interpersonal trust in a community and feelings of trust, safety, and reciprocal relationship, which Harpham and colleagues refer to as "cognitive measures" (29). In addition, "structural" variables have been used to define and measure social capital, including the level of volunteerism, organizational membership or participation, civic engagement, and links to groups with resources both within and outside of a community (21,26,29). Potential indicators of social cohesion include the strength of social networks, connections, and interpersonal trust. Potential indicators of social capital that could be compared across socioeconomic environments include the number and density of community organizations, volunteerism or participation in voluntary organizations, voter registration, and voter turnout.

## State of the Metrics

### Community socioeconomic composition

A valuable source of data on socioeconomic indicators is the US decennial census. Using census data has many benefits; specifically, the data are publicly available and can be compiled for many units of geography, including the block level, tract level, zip code, county, and other defined areas. Nonetheless, census data also have limitations; the data are only collected every 10 years, census units or boundaries change over time, and many measures are sensitive to migration in and out of communities. In addition, a person's census tract or other geographic unit is not necessarily his or her socioeconomic environment (30). Identifiable "neighborhoods" do not always correspond to administratively determined units of geography, such as census tracts or zip codes.

Another useful resource is the American Community Survey (ACS), which is a key part of the Census Bureau's efforts to revamp and expand the decennial census program. The ACS is a random sample, population-based survey of counties designed to produce demographic, economic, social, and housing information more often than every 10 years. The ACS started in selected counties in 1996 and expanded in 2005 to include all US counties, the District of Columbia, and 78 municipalities in Puerto Rico. Beginning in 2005, the ACS produced 1-year estimates of key variables for geographic areas with 65,000 people or more. In 2008, the ACS released 3-year estimates of these indicators for areas with 20,000 people or more. For areas with populations of less than 20,000, 5-year estimates based on data from 2005 to 2009 will be released after 2010. As with the decennial census, response to the ACS questionnaire is required by law. Most socioeconomic indicators can be obtained from the ACS at the county level.

As part of the federal initiative No Child Left Behind, states are required to collect and report yearly program statistics for public school systems. District- and school-level statistics regarding graduation rates and student performance in reading and math can be accessed at www.schooldatadirect.org, which is maintained by the nonprofit Council of Chief State School Officers. More detailed information can also be accessed through state agencies charged with collecting and maintaining the data.

The data collected by the census, the ACS, and No Child Left Behind offer economic and educational indicators that are publicly available for measurement at the county level (and for smaller units) over time. Although it is possible to stratify these indicators by race and ethnicity to assess disparities, the necessary data are not publicly available and such analyses would be labor-intensive.

### Social structure

Income inequality can be measured with data on per capita or household income in a geographic area, which are readily available from the census. Approaches used to operationalize the measurement of income inequality include 1) the Gini coefficient, which is a measure of the statistical dispersion of income or wealth in a population, ranging on a standardized scale from 0 (perfect equality or everyone has the same amount of money) to 1 (perfect inequality; 1 person has all the income and everyone else has none); and 2) the Robin Hood index (also called the Pietra ratio), the proportion of income that has to be transferred from those above the mean to those below to create an equal distribution (19-21). Kawachi and Kennedy found that the association between income inequality in US states and mortality rates did not vary across 6 measures of income distribution (31).

Residential racial segregation can be measured reliably with census data (22). Segregation is typically measured by using the "index of dissimilarity," which indicates the evenness with which 2 groups are distributed across component geographic units (eg, census tracts) of a larger area (eg, county or metropolitan statistical area), or using the Gini coefficient (21).

Discrimination reflects social structure, which refers to the enduring social relationships, norms, and patterns of behavior within a society. Discrimination is difficult to measure both in the cross-section and over time, and it is virtually impossible to measure at a contextual level (23,24). Researchers typically rely on self-reports of perceived harassment and discrimination both within and outside of respondents' community context. The methods used to measure perceived discrimination have varied extensively; this type of data is not readily available across communities.

### Social cohesion or social capital

Many population-based surveys and individual research projects have attempted to measure neighborhood social cohesion and the benefits (or social capital) that can result. For example, both the Project on Human Development in Chicago Neighborhoods and the Los Angeles Family and Neighborhood Study use multi-item scales of social cohesion (15). Unfortunately, metrics for this area are not well developed (26). There is no agreed-upon approach for measuring social or community cohesion, and no data are available across time and communities (29).

A reasonable measurement strategy for social capital that can be applied consistently across many contexts is the structural approach, which focuses on community engagement and civic participation. Community engagement can be measured by the number and density of community and voluntary organizations in a defined geographic area and by the participation level of community members in these organizations. In addition, voter registration and participation can serve as markers for civic engagement. Basic voter registration information is published by the Census Bureau every election year but not at the local level. The Help America Vote Act of 2002 mandates that states establish a database of registered voters, but these systems are not yet available for use. The best information currently available comes from private firms.

Data on voter turnout are available from the US Election Assistance Commission (EAC), updated every 2 years after congressional and presidential elections. State-level data are available to the public through the EAC Web site, and more detailed data are available to approved researchers. In addition, access to the EAC's records can be requested under the Freedom of Information Act.

## Recommendations

Identifying a set of indicators for the socioeconomic environment on which incentives for population health can be based is a worthwhile yet daunting task, especially given the methodological and measurement challenges to research attempting to establish causal links between multiple nonrandom social and economic exposures and health outcomes. Considering the state of the research, the current state of data and metrics for health outcome variables at the local level, and the potential for program and policy intervention, we rank the following set of indicators as potentially powerful in assessing and motivating communities' progress toward population health goals, both in the medium term (3-5 years) and beyond:

1. Poverty rate
2. Unemployment rate
3. Average household income
4. Affordability of single-family home
5. Bankruptcy and foreclosure rates
6. Percentage of households on public assistance
7. Percentage of single-parent households
8. Percentage of children receiving free or reduced-price lunch
9. Concentrated disadvantage and concentrated affluence scales
10. Percentage of adults older than 24 years with less than a high school education
11. Percentage of adults older than 18 years with less than an eighth-grade education
12. Public high school graduation and dropout rates
13. Percentage of third- and tenth-grade students at grade level in reading
14. Percentage of tenth-grade students at grade level in math
15. Racial segregation
16. Density of voluntary organizations
17. Voter registration and turnout

The broad list of indicators in this article is consistent with the recommendations of numerous researchers and opinion leaders regarding investments related to the social determinants of health (14,32). Population health improvements depend on improvements in many of the fundamental social determinants of health including educational opportunities, safe and meaningful employment, income security, and engaged, active communities free from poverty and discrimination. Despite serious limitations and challenges in the science and the state of many of the metrics proposed here, further investments in such development are critical to efforts to measure, promote, and achieve population health.
